# Biomechanical Gain in Joint Excursion from the Curvature of the Achilles Tendon: Role of the Geometrical Arrangement of Inflection Point, Center of Rotation, and Calcaneus

**DOI:** 10.3390/diagnostics11112097

**Published:** 2021-11-12

**Authors:** Ryuta Kinugasa, Naoto Yamamura, Shu Takagi, Shantanu Sinha

**Affiliations:** 1 Department of Human Sciences, Kanagawa University, Yokohama 221-8686, Japan; 2 Department of Mechanical Engineering, The University of Tokyo, Bunkyo-ku, Tokyo 113-8656, Japan; nyama@g.ecc.u-tokyo.ac.jp (N.Y.); takagi@mech.t.u-tokyo.ac.jp (S.T.); 3 Muscle Imaging and Modeling Laboratory, Department of Radiology, University of California San Diego, San Diego, CA 92121, USA; shsinha@health.ucsd.edu

**Keywords:** ankle geometrical model, ankle plantarflexion, calcaneus position, gear, joint kinematics

## Abstract

The dorsal movement of the Achilles tendon during ankle rotation is restricted by anatomical obstructions. Previously, we demonstrated that the anatomical obstruction provides a gain (gain_AT_) in the proximal displacement of the calcaneus compared to the change in the Achilles tendon length. Here, we empirically validate and extend our previous modeling study by investigating the effects of a broad range of obstruction locations on gain_AT_. The largest gain_AT_ could be achieved when the obstruction was located on the most ventral and distal sides within the physiological range of the Achilles tendon, irrespective of the ankle position.

## 1. Introduction

The movement of the foot during ankle plantarflexion is amplified compared with the shortening of the plantarflexor muscles. This amplification translating the muscle fascicle shortening into joint action, termed as a “gain”, is calculated as the ratio of the magnitude of the calcaneus movement to that of muscle fascicle shortening. The gain was reported to be typically higher than 2.3 [1]. Several mechanisms have been proposed to explain the gain. The first mechanism considers the oblique orientation of the pennate muscle fibers. For instance, Gans [2] suggested that a pennate fiber arrangement between the aponeuroses, which remain at a constant distance from each other during contraction, results in a movement of the aponeurosis parallel to the long axis of the muscle, which is amplified compared with the shortening of the muscle fiber. This is due to fascicle rotation by a factor equal to the inverse of the cosine of the pennation angle. The second mechanism considers the mechanical constraints on the lateral movement (i.e., moving closer or further away) of the aponeuroses. In particular, variations in the aponeurosis separation (i.e., muscle thickness) before contraction varies the gain such that the aponeuroses moving closer together decrease the gain, and those moving further apart increase the gain [3], a mechanism that becomes important during muscle atrophy that accompanies aging. However, these two effects are insufficient to completely account for the overall amplification.

This discrepancy in the total amplification could be explained by a third mechanism involving the mechanical constraint that prevents the outward (i.e., dorsal) movement of the Achilles tendon, causing the tendon to bend. Hodgson et al. [3] first introduced the concept of a rigid restriction on the dorsal movement of the Achilles tendon due to the presence of a fixed inflection point or bend in the tendon during ankle plantarflexion. They demonstrated through simulation that this inflection point or bend in the tendon results in an amplification of the proximo-distal displacement of the calcaneus (the distal endpoint of the Achilles tendon) relative to the myotendinous junction between the soleus muscle and the Achilles tendon (proximal start point of the Achilles tendon). This gain from the Achilles tendon (gain_AT_), which is defined as the ratio of the vertical displacement of the calcaneus to the change in the Achilles tendon length, varies depending on both the inflection point with respect to the ankle center of rotation (COR) and the ankle position. In fact, Hodgson et al. [3] evaluated the gain_AT_ at three locations of the inflection point (Supplementary Materials Figure S1) given by coordinates (*x*, *y*; horizontal and vertical axes of a system of coordinates, in cm) and obtained the largest gain_AT_ for an inflection point located at (3, 0) from ankle COR at (0, 0). This was followed by a decreasing order of gain_AT_ observed at inflection points located at (4, 0) and (3, 2.5) during ankle plantarflexion. In contrast, the gain_AT_ decreased as the ankle dorsiflexed. Several significant issues posed in this previous report include the fact that the gain_AT_ is unknown at locations other than the three locations mentioned above, and the geometrical structure of the actual human musculoskeletal system is neglected. Further, the mechanisms determining the gain_AT_ are still not well understood, and the location resulting in the largest gain_AT_ remains unknown.

Therefore, we extended a previous modeling study [3] from our research group by performing direct measurements of the physiological range of the inflection point within the Achilles tendon and the ankle position for in vivo humans using magnetic resonance imaging (MRI). We investigated the effects of a broad range of inflection point locations on the gain_AT_ during ankle rotation at the two positions. The present study conducted a modeling investigation, because it was difficult to identify the location of the inflection point within the Achilles tendon that exhibits the largest gain_AT_ within the physiological range of the Achilles tendon using an in vivo human experimental study.

## 2. Methods

**Figure 1:**
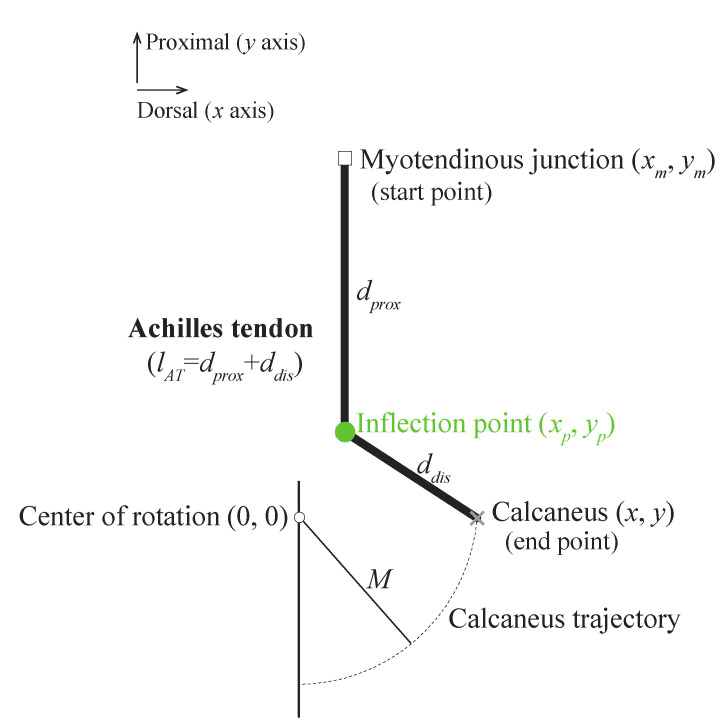
Simplified ankle model with the presence of an inflection point. The inflection point prevents the Achilles tendon from moving dorsally. The arc indicates the trajectory of the calcaneus during ankle joint rotation. Radius M is the distance from the ankle center of rotation to the insertion of the Achilles tendon on the calcaneus (i.e., inferior calcaneal tuberosity, grey cross symbol). Distance $d_{dis}$ is the length of the segment from the inflection point to the insertion of the Achilles tendon on the calcaneus, whereas $d_{prox}$ is the length of the segment from the myotendinous junction to the inflection point. The Achilles tendon length $l_{AT}$ is defined as the sum of the distances from the myotendinous junction to the inflection point and from the inflection point to the calcaneus.

We used the simplified ankle model (Figure 1) based on the quadratic solution from Appendix B in [3]. The movement of the calcaneus around the ankle center of rotation is indicated by the dotted black arc with radius M. The Achilles tendon is attached to the calcaneus but is prevented from anterior motion by an inflection point fixed at coordinates $x_{p}$, $y_{p}\;$ from the ankle center of rotation. This model helped to understand the effects of the locations of the inflection point on gain_AT_ over a range of ankle positions.

The gain_AT_ is defined as the reciprocal of the change in the Achilles tendon length ($\Delta l_{AT}$) when the calcaneus is displaced by 1 mm, as follows:

(1) $${\mathrm{gain}}_{\mathrm{AT}}=\frac{1}{\Delta l_{AT}}$$

The Achilles tendon length ($l_{AT}$) is defined as follows:

(2) $$l_{AT}=d_{prox}+d_{dis}$$

where $d_{prox}\;$ and $d_{dis}\;$ are the distances from the myotendinous junction to the inflection point and from the inflection point to the Achilles tendon insertion on the calcaneus, respectively. They are given as follows:

(3) $$d_{prox}=\sqrt{{\left(x_{p}-x_{m}\right)}^{2}+{\left(y_{p}-y_{m}\right)}^{2}}$$

(4) $$d_{dis}=\sqrt{{\left(x-x_{p}\right)}^{2}+{\left(y_{p}-y\right)}^{2}}$$

where the coordinates (x, y), ($x_{p}$, $y_{p}$), and ($x_{m}$, $y_{m}$) represent the positions of the Achilles tendon insertion on the calcaneus, inflection point, and myotendinous junction, respectively.

**Figure 2:**
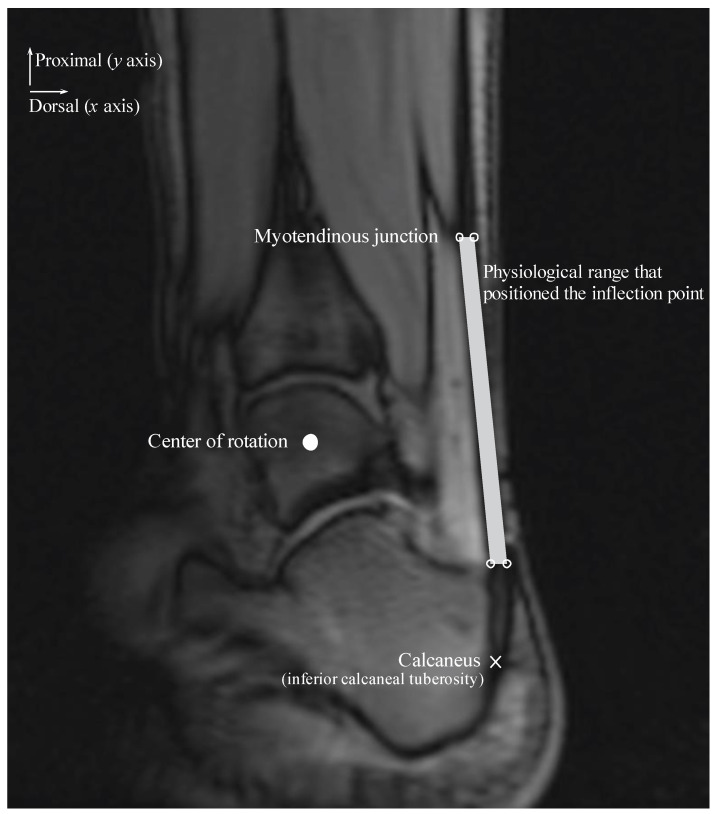
Representation of the physiological range of the inflection point within the Achilles tendon where the gain_AT_ is calculated. The positions of the start (i.e., myotendinous junction) and end (i.e., inferior calcaneal tuberosity) points of the Achilles tendon, and ankle center of rotation are shown in a typical sagittal-plane MR image from one subject. The ankle angle was set at approximately –10° (ankle dorsiflexed position). The physiological range of the inflection point is defined as the area within the Achilles tendon and is represented as the area (gray color) of a quadrilateral comprising four vertices (points; indicated by white circle symbols).

We considered different coordinates for the inflection point within a physiological range along the *x* and *y* axes during the ankle joint rotation (Figure 2). The physiological range of the Achilles tendon was defined as the area within the Achilles tendon and represented the area of a quadrilateral comprising four sides and four vertices. The upper right and left points are on the same horizontal line as the myotendinous junction location and are positioned on the most dorsal and ventral edges of the Achilles tendon. The lower right and left points are on the same horizontal line as the most distal end of the Kager’s fat pad and are positioned on the most dorsal and ventral edges of the Achilles tendon.

Signed and informed consent was attained to participate in an Institutional Review Board-approved study. The physiological range of the Achilles tendon was determined using velocity-encoded phase-contrast MRI as the ankle was rotated with a hydraulically operated foot-pedal device. The details of this imaging technique and device have been previously described [4]. MR images were acquired from three subjects (age: 26.7 ± 6.4 years, height: 166.3 ± 4.0 cm, weight: 57.7 ± 8.7 kg; two men and one woman) with a 1.5-Tesla MR scanner (GE Healthcare, Milwaukee, WI, USA). Velocity encoding of 10 cm/s in the superior-inferior direction was used, with three views per segment, repetition time of 13.3 ms, echo time of 7.5 ms, flip angle of 20°, 3-mm slice thickness, 290-Hz receiver bandwith/pixel, 128 × 256 matrix size, 160 × 320 mm field of view (FOV), and two averages. Each subject was instructed to fully relax as the ankle was passively plantarflexed and dorsiflexed using the pedal device. The pedal device was made up of a computer-controlled, servomotor driven “master” piston-cylinder (placed outside the magnet room) that enabled pumping of hydraulic fluid in a prescribed/programmed cyclical manner, through high-pressure tubes to a “slave” non-magnetic piston-cylinder inside the magnet bore that in turn rotated the foot pedal. The pedal device was programmed to complete a rotational cycle of 30°, from 20° (ankle plantarflexed position) to −10° (ankle dorsiflexed position).

The physiological range of the Achilles tendon was measured at the ankle plantarflexed and dorsiflexed positions and subsequently averaged for the three subjects for each ankle position. Additionally, the insertion positions of the Achilles tendon on the calcaneus and COR of the ankle were measured. The position of the Achilles tendon insertion on the calcaneus was displaced along an arc of radius M centered at the ankle COR with 1 mm increments in the calcaneus displacement in plantarflexed and dorsiflexed positions. We assumed that the $d_{prox}$ remained constant during each calcaneus displacement. Thus, $\Delta l_{AT}$ could be calculated as follows:

(5) $$\Delta l_{AT}=d_{dis+1}-d_{dis}$$

where $d_{dis}$ and $d_{dis+1}\;$ denote the distance from the inflection point to the calcaneus in a given ankle position and when the calcaneus position changes by 1 mm, respectively. The moment arm was calculated as the shortest perpendicular distance between the COR and the line connecting the inflection point to the calcaneus.

All values are presented as the mean and standard deviations.

## 3. Results

**Figure 3:**
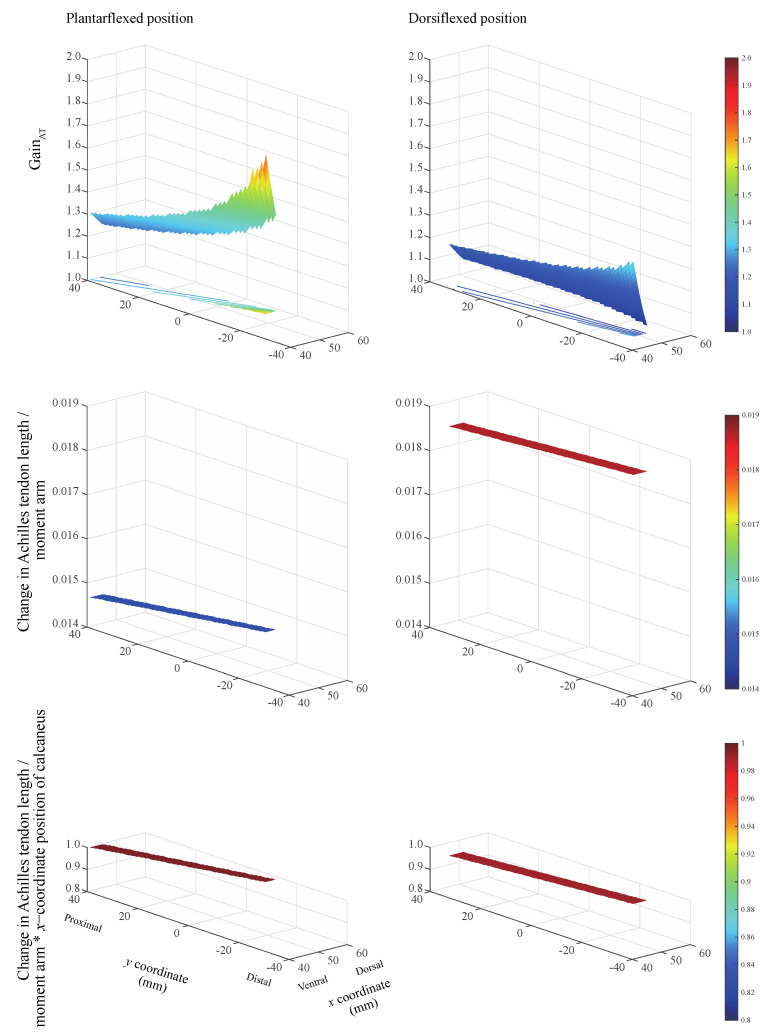
Effects of inflection point locations and ankle positions on gain_AT_ (**top**), ratio of change in the Achilles tendon length to moment arm (**middle**), and product of the ratio of change in the Achilles tendon length to moment arm and the *x*-coordinate of calcaneus position (**bottom**), for 1 mm calcaneus displacement. We used a model where the position of the inflection point is varied, whereas the displacements of the myotendinous junction with respect to the calcaneus remains constant. Three-dimensional surface plots showing the change in the Achilles tendon length (Z axis) with respect to the location of the inflection point along the *x* (X axis) and *y* (Y axis) directions. This change is shown for two ankle positions: plantarflexed (**left**) and dorsiflexed (**right**) positions.

The gain_AT_ always exceeded unity when the inflection point was located within the physiological range for the two ankle positions (Figure 3, top). This indicates that the calcaneus displacement has a gain corresponding to the change in the Achilles tendon length. The largest gain_AT_ was realized when the inflection point is located on the most ventral and distal sides, irrespective of ankle position. The ratio of the change in the Achilles tendon length to the moment arm remains nearly constant (Figure 3, middle). The product of the ratio of the change in the Achilles tendon length to the moment arm and the *x*-coordinate of the calcaneus position remains nearly constant within the physiological range of the Achilles tendon. This value equaled 0.990 and 0.996 when the ankle remained in the plantarflexed and dorsiflexed positions, respectively (Figure 3, bottom). The ankle angle and Achilles tendon insertion on the calcaneus *(x, y)* coordinates were measured as 20.7 ± 1.5° and (*x* = 67.8 ± 7.1, *y* = −34.4 ± 4.2) mm at the plantarflexed position, and −9.3 ± 0.8° and (*x* = 52.7 ± 9.9, *y* = −55.0 ± 2.6) mm at the dorsiflexed position.

When using the proposed model, any change in the inflection-point position within the physiological range of the Achilles tendon, while displacing the calcaneus relative to the displacement of the myotendinous junction, causes the ratio of the change in the Achilles tendon length to the moment arm to remain nearly constant. When the ratio of the change in the Achilles tendon length to the moment arm was multiplied by the *x*-coordinate position of the calcaneus, the value was approximately 1. These results indicate that gain_AT_ can be estimated using the moment arm and the *x*-position of the calcaneus (calcaneus_x) as follows:

(6) $${\mathrm{gain}}_{\mathrm{AT}}≅\frac{\mathrm{calcaneus}\_x}{\mathrm{Moment}\;\mathrm{arm}}$$

Accordingly, the maximum relative error between gain_AT_ calculated considering the change in the Achilles tendon length (Equation (1)) and that calculated using the moment arm (Equation (6)) equals 0.38% and 0.98% in the plantarflexed and dorsiflexed positions, respectively.

## 4. Discussion

We evaluated the effects of a broad range of inflection point locations on the gain_AT_, during ankle rotation at two ankle positions. The largest gain_AT_ was observed when the inflection point is located on the most ventral and distal sides within the physiological range. However, unexpectedly, this was independent of the ankle position. The former result is observed because as the gain_AT_ increases and the inflection point is closer to the ankle COR. These results are not consistent with those presented by Hodgson et al. [3], who demonstrated that as the gain_AT_ increases, the inflection point is closer to the ankle COR. This difference might be partly due to the different models of the inflection point and ankle position employed. While our model relied on human data obtained empirically from physiological experiments, Hodgson et al. [3] established their model on logical speculation; thus, it was not based on human data. The results of our model are partially supported by the findings of the experimental investigation conducted by Csapo et al. [5], who demonstrated that there was no significant difference in the gain_AT_ among passive ankle rotations from 10° to 20°, 0° to 10°, and −10° to 0°.

Gain_AT_ is not determined solely by the distance from the ankle COR to the inflection point but rather by the geometrical arrangement of the inflection point, myotendinous junction, and ankle position relative to the ankle COR. Although the geometry of the ankle COR and ankle position is determined by an intrinsic arrangement, the location of the inflection point can be variable. Our previous experimental and computational investigations using a multi-modality approach revealed that the inflection point of the Achilles tendon is due to neither the nature of the tissue deformations surrounding the Achilles tendon nor its physical properties [4]. Instead, we concluded that the inflection point results from the geometric architecture of the Achilles tendon and its configuration relative to the surrounding tissues, such as the Kager’s fat pad. This pad is a mass comprised of adipose tissue, the synovial membrane located in the superior tuberosity of the calcaneal bone inferiorly, and the Achilles tendon posteriorly [6]. As the ankle plantarflexes, the calcaneus moves proximally and dorsally, and a force is produced in the ventral direction around the inflection point location. These configurations appear to deform the Kager’s fat pad, the degree of which is determined by the stiffness of the Kager’s fat pad. Because this pad is comprised of adipose cells [7], such deformation is assumed to minimize pressure change during ankle flexion [8]. Surgery [9], aging [10], and diabetic disease [11] alter the stiffness of the fat pad due to fibrosis, resulting in changes in the location of the inflection point, and ultimately, in the gain_AT_.

The importance of the gain mechanism, amplifying muscle fascicle shortening to a larger calcaneus movement [1], has been recognized in terms of the action of skeletal lever systems [12], with a gain higher than 2.3 being observed in such systems. For explaining such gains completely, note that the fiber architectural and aponeurosis movement effects could increase the gains by 0.04 to 0.50 for pennation angles from 15 to 50°, and by 0.08 to 0.33 for aponeurosis separations from 14.9 to 18.9 mm, respectively [3]. If the remaining gain (1.47~2.18) is because of the mechanism of the inflection point, the change in the Achilles tendon length is required to be in the range of 0.46 to 0.68 mm relative to 1 mm calcaneus displacement. Our findings reported herein indicate that this condition can be sufficiently satisfied by the inflection point location. Our results highlight a previously unappreciated occurrence of gain mechanism that occurs at the level of the Achilles tendon, and they explain the apparent discrepancy between the change in the muscle fascicle length and the calcaneus displacement.

Individuals with pronounced tendon curvature may be at a higher risk of Achilles tendon rupture. A smaller change in the Achilles tendon length relative to the moment arm at plantarflexion than that at dorsiflexion is demonstrated in Figure 3 (middle). This suggests a short moment arm in extreme plantarflexion that translates to higher tendon loading. Our modeling investigation revealed that the constraint to the posterior movement of the Achilles tendon due to a presence of inflection point reduces the moment arm at the ankle over a wide range of ankle angles [13]. Thus, people with highly curved tendons experience higher tendon loads, resulting in a higher risk of Achilles tendon ruptures.

The limitation of this study is associated with the measurement of the COR. The selection of the sagittal image strongly influenced the determination of the COR. The talocrural joint axis was not orthogonal to the sagittal plane; it deviated by approximately 15° from the transverse plane and by approximately 20° from the coronal plane [14]. Further, the shape of the bone depicted in the imaging plane could be altered as a function of the joint movement [15]. These may be potential factors inducing errors in the determination of the COR. Another limitation in the present study was the determination of the physiological range of the position of the inflection points determined in the case of the three volunteers. Further, it is well established that there is gender difference in anthropometric parameters of the Achilles tendon while there is no racial difference [16]. In contrast, a biomechanical study of the viscoelastic properties of the Achilles tendon complex showed higher stiffness in black athletes [17]. Future studies need to be conducted with diverse ranges of subjects.

In conclusion, as a result of our model study investigating the relationship between the broad range of location of the inflection point and the degree of gain_AT_, the largest gain_AT_ can be realized when the inflection point is located on the most ventral and distal sides within the physiological range of the Achilles tendon regardless of the ankle position. Thus, this study affords a better understanding of the possible factors influencing the gain_AT_, the evaluation of which might be essential to accurately predict the joint kinematics generated by large muscle-tendon complexes.

## Data Availability

The datasets generated and analyzed during the current study are available from the corresponding author on reasonable request.

## Supplementary Materials

The following are available online at https://www.mdpi.com/article/10.3390/diagnostics11112097/s1, Figure S1: The simplified model used to calculate the gain_AT_ with respect to the three positions of the inflection point relative to the ankle center of rotation (Hodgson et al. 2006).
