# Supplementary material for: Biomechanical Gain in Joint Excursion from the Curvature of the Achilles Tendon: Role of the Geometrical Arrangement of Inflection Point, Center of Rotation, and Calcaneus
Source: Diagnostics (Basel). 2021 Nov 12;11(11):2097. doi: 10.3390/diagnostics11112097 (PMC8618986; doi:10.3390/diagnostics11112097)
Supplement: Supplementary file 1 [file diagnostics-11-02097-s001.zip › diagnostics-1450805-SI.pdf]

diagnostics MDPI

**SUPPLEMENTARY FIGURE**

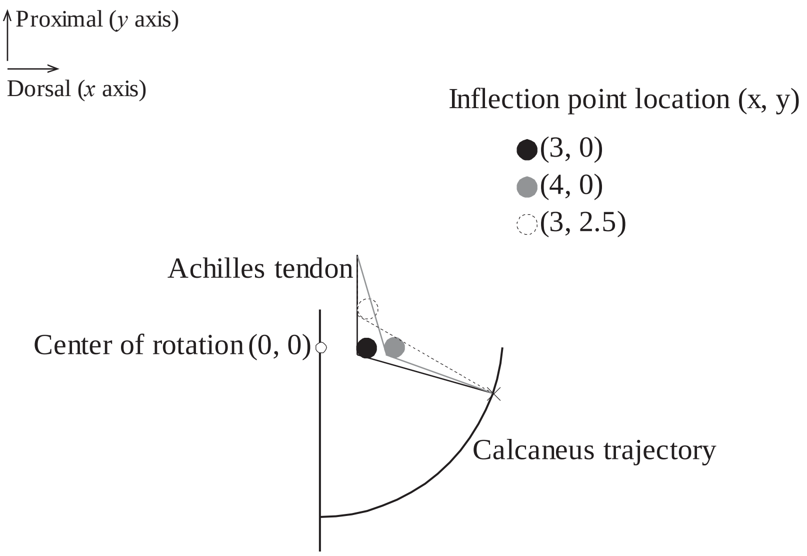


**Supplemental Figure S1.** The simplified model used to calculate the $gain_{AT}$ with respect to the three positions of the inflection point relative to the ankle center of rotation (Hodgson et al. 2006). The movement of the calcaneus around the ankle center of rotation is indicated by the black arc. The Achilles tendon is attached to the calcaneus but is prevented from moving anteriorly by a fixed inflection point at coordinate x, y from the ankle center of rotation.
